# Normal ex vivo mesenchymal stem cell function combined with abnormal immune profiles sets the stage for informative cell therapy trials in idiopathic pulmonary fibrosis patients

**DOI:** 10.1186/s13287-021-02692-0

**Published:** 2022-01-31

**Authors:** Elena Atanasova, Dragana Milosevic, Svetlana Bornschlegl, Karen P. Krucker, Eapen K. Jacob, Eva M. Carmona Porquera, Dagny K. Anderson, Ashley M. Egan, Andrew H. Limper, Allan B. Dietz

**Affiliations:** 1 Department of Laboratory Medicine and Pathology, Mayo Clinic College of Medicine, Rochester, MN USA; 2 Department of Laboratory Medicine and Pathology, Divisions of Clinical Biochemistry and Immunology, Mayo Clinic College of Medicine, Rochester, MN USA; 3 Division of Transfusion Medicine, Mayo Clinic, Rochester, MN USA; 4 Thoracic Diseases Research Unit, Division of Pulmonary Critical Care and Internal Medicine, Mayo Clinic College of Medicine, Rochester, MN USA; 5 Divisions of Transfusion Medicine and Experimental Pathology, Immune Progenitor and Cell Therapeutics (IMPACT) Lab, Mayo Clinic College of Medicine, Rochester, MN USA

**Keywords:** Mesenchymal stem cells, Idiopathic pulmonary fibrosis, Therapy, Immunology

## Abstract

**Background:**

Idiopathic pulmonary fibrosis (IPF) is a chronic, progressive pulmonary disease characterized by aberrant tissue remodeling, formation of scar tissue within the lungs and continuous loss of lung function. The areas of fibrosis seen in lungs of IPF patients share many features with normal aging lung including cellular senescence. The contribution of the immune system to the etiology of IPF remains poorly understood. Evidence obtained from animal models and human studies suggests that innate and adaptive immune processes can orchestrate existing fibrotic responses. Currently, there is only modest effective pharmacotherapy for IPF. Mesenchymal stem cells (MSCs)-based therapies have emerged as a potential option treatment of IPF. This study characterizes the functionality of autologous MSCs for use as an IPF therapy and presents an attempt to determine whether the disease occurring in the lungs is associated with an alterated immune system.

**Methods:**

Comprehensive characterization of autologous adipose-derived MSCs (aMSCs) from 5 IPF patient and 5 age- and gender-matched healthy controls (HC) was done using flow cytometry, PCR (ddPCR), multiplex Luminex xMAP technology, confocal microscopy self-renewal capacity and osteogenic differentiation. Additionally, multi-parameter quantitative flow cytometry of unmanipulated whole blood of 15 IPF patients and 87 (30 age- and gender-matched) HC was used to analyze 110 peripheral phenotypes to determine disease-associated changes in the immune system.

**Results:**

There are no differences between autologous aMSCs from IPF patients and HC in their stem cell properties, self-renewal capacity, osteogenic differentiation, secretome content, cell cycle inhibitor marker levels and mitochondrial health. IPF patients had altered peripheral blood immunophenotype including reduced B cells subsets, increased T cell subsets and increased granulocytes demonstrating disease-associated alterations in the immune system.

**Conclusions:**

Our results indicate that there are no differences in aMSC properties from IPF patients and HC, suggesting that autologous aMSCs may be an acceptable option for IPF therapy. The altered immune system of IPF patients may be a valuable biomarker for disease burden and monitoring therapeutic response,

**Supplementary Information:**

The online version contains supplementary material available at 10.1186/s13287-021-02692-0.

## Background

Idiopathic pulmonary fibrosis (IPF) is a complex disorder caused by multiple injuries to lung epithelium which triggers a local immune response leading to dysregulation of cellular homoeostasis [1]. Accumulations of extracellular matrix and scar formation in IPF are consequences of impaired wound repair mechanisms [2–4]. The pathogenesis is still poorly understood, and with the exception of lung transplantation, currently there are no significantly effective pharmacotherapies for IPF [2, 5–8]. Growing body of evidence from basic science and translational research indicates that IPF appears to be a direct result of immune dysregulation and aberrant wound-healing response in the lungs [2, 9, 10]. Not much is known about the contribution of the immune system to the development of IPF or how lung immune responses affect the systemic immunity. To the best of our knowledge there are no studies, which comprehensively investigate the immune status of IPF patients, although there are few studies identifying potential leukocytes involved with IPF pathogenesis [8, 11–17].

MSCs are important regulators of tissue repair and wound-healing processes, have anti-inflammatory properties and display significant immunomodulatory capacity [18]. The use of MSCs-based therapy has emerged as a potential option for treatment of IPF. We have developed an expansive clinical program [21–25] evaluating the therapeutic index of autologous and allogeneic MSC in a variety of conditions, none of which have evaluated patients with chronic progressive fibrosis. There are promising results of preclinical [24–26] and clinical studies [27–30] using allogeneic MSC in assessing their safety for IPF treatment. However, the use of autologous adipose MSCs has not being investigated as a possible treatment of IPF.

This study explores the suitability of autologous adipose MSCs as a viable therapy for IPF. Furthermore, it is an attempt to determine whether the disease occurring in the lungs can be reflected on peripheral blood immune status and thus be used to stage the disease as well as monitoring/predicting changes during MSC therapy.

## Materials and methods

### Procedural factors

IPF patients (group of 5) were identified from Interstitial Lung Diseases Outpatient Clinic by an expert pulmonologist in IPF and other fibrotic diseases of the lung following the ATS/ERS/JRS/ALAT Statement criteria [31]. All the aspects of this study involving samples from IPF patients and age- and gender-matched healthy volunteers were reviewed and approved by the Mayo Clinic Institutional Review Board. All subjects provided written informed consent to participate.

**Table:** Patients characteristics for adipose tissue collection and MSC isolation

Variable	Characteristics	IPF (*n* = 5)	Healthy control (*n* = 5)
Age (years)	Mean	77.8	74.8
	Range	73–80	61–80
Gender	Male	3	3
	Female	2	2

### Isolation, propagation and identification of adipose MSC (aMSC)

Abdominal wall adipose tissue (approximately 1.5–2.5 g) was obtained under sterile conditions from IPF patients and age- and gender-matched healthy donors in an outpatient surgical suite. Tissues were processed within 2 h of procurement. Cells were expanded ex vivo according to the protocol based on Standard Operation Procedures for isolation, extraction and expansion of aMSC analogs for clinical use [32, 33]. In brief, after microdissection the fat tissue was digested with collagenase Type I at 0.075% w/v, (Worthington Biochemicals, Lakewood, NJ) for 1.5 h at 37 °C. Adipocytes were separated from the vascular fraction by centrifugation (400×*g* for 5 min, at room temperature). The cell pellet was washed with PBS and passed through cell strainers (70 μm followed by 40 μm, BD Biosciences, Franklin Lakes, New Jersey). The resulting cell fraction was plated in T-75 cm^2^ flasks (Thermo Fisher, Waltham, MA) and incubated in a fully humidified incubator supplied with 5%CO_2_ in PLGold xeno-free media. The xeno-free media named “PLGold media” consist of: Advanced MEM (Thermo Fisher Scientific) supplemented with 5% (v/v) PLTGold, (Mill Creek Life Sciences, Rochester, MN), 1% (v/v) GlutaMax (Thermo Fisher) and 1% (v/v) antibiotics (100 U/ml penicillin, 100 g/ml streptomycin, HyClone, Logan, UT). Cells were propagated when they were 60–80% confluent using TrypLE (Trypsin-like Enzyme, Invitrogen, Carlsbad, CA) [30]. Cells yield and viability was quantified using acridine orange (*AO)* and propidium iodide *(*PI*)* nuclear stains for exclusion assay on Luna-FL Dual Fluorescence Cell Counter (all from Logos Biosystems, Annandale, VA). All aMSC used in the experimental procedures were between passages 2 and 5.

The proliferation and growth rate of aMSCs was monitored by adding IncuCyte NucLight Rapid Red Reagent for Nuclear Labeling at 1:500 dilution (Essen Bioscience, Ann Arbor, MI) in the media. After 30-min incubation at 37 °C in a fully humidified incubator supplied with 5%CO_2_ cells were placed in IncuCyte S3 Live Cell Analysis instrument (Sartorius, Ann Arbor, MI) for fluorescent quantification of cell proliferation. Fluorescent images of red nuclei from sixteen fields in each well were captured at 681 nm every 6 h with 10 × objective. Each cell count was repeated twice in four replicas. The data acquisition, visualization and analysis were done using internal IncuCyte S3 Analyzing Software. The growth rate kinetic and doubling times (*t*_*d*_) were evaluated recording the cell proliferation rate by counting the number of red nuclei every six hours for duration of five days. The population doubling time (*t*_*d*_) was calculated by computing the linear regression of *N* = *N*_0_ × *e*^*kt*^, where *N* is the number of red nuclei count at time *t*, *N*_0_ is red nuclei count at time *t* = 0, and *k* is cell growth rate per hour.

Cell phenotype was analyzed by labeling them with primary fluorochrome-conjugated monoclonal antibodies, as described before [32, 34, 35]. Samples were analyzed using the Beckman Coulter Gallios 3-laser, 10-color flow cytometer and Kaluza 2.1 software (Beckman Coulter, Chaska, MN).

## Morphologic characterization

Cells were seeded at 6100 cells/cm^2^ in a sterile eight well chamber (Cellvis, Sunnyvale, CA) for 48 h. Fresh media containing 350 nM MitoTracker Red CXMRos (Invitrogen) were added to the cells and incubated 30 min in a fully humidified incubator supplied with 5%CO_2_, followed by washing with PBS. Cells were fixed by adding 10% buffered formalin (Azer Scientific, Morgantown, PA). The covered glass chambers wrapped in aluminum foil were kept for 30 min on a rocker at room temperature. PBS-washed cells were permeabilized with 0.3% TRITON X-100 in PBS containing Hoechst 33342 (1:1000 dilution, Thermo Fisher) and AlexaFluor™ 488 Phalloidin (1:1500 dilution, Thermo Fisher). Aluminum foil-wrapped covered glass chambers were kept for 30 min on a rocker at room temperature. Cells were washed with PBS and kept in PBS to prevent them from drying during imaging.

2D and 3D cell images were collected using a laser scanning confocal microscope LSM 780 and ZEN 2010 software (Carl Zeiss, NY). For quantification of the cells mitochondrial volume five individual aMSC from each cell line [total 25 cells from healthy controls (HCaMSC) and 25 cells from IPF patients (IPFaMSC)] were imaged under the same acquisition conditions: image size (512 × 512 pixels), number of Z-stack slices (16 slices, 7.692 μm), number of averaging Z-stuck slices (averaging 2 Z-stuck slices), scan zoom (X: 1.0, Y: 1.0), pinhole sizes and laser intensities (1.42 AU for 405-nm laser at 50% intensity, 1.19 AU for 488-nm laser at 50% intensity and 0.99 AU for 561-nm laser at 40% intensity) using C-Apochromat 63x/1.2 W Korr objective.

Detection was carried out at wavelengths of 406–480 nm for Hoechst 33342 (nuclear stain), 499–560 nm for AlexaFluor™ 488 Phalloidin (actin F stain) and 566–696 nm for MitoTracker Red CXMRos (mitochondria stain). For image analysis and mitochondria volume calculations Image Data Management Software Imaris 8 (Oxford Instruments, Abingdon, GB) was used. Unpaired Student's t test analysis was used to determine the statistical difference in mitochondria volumes between aMSC of the two tested groups.

## Adipogenic differentiation capacity

Cells (passages 2 and 3) previously cultured in PLGold media were cultured for two consecutive passages in MSC NutriStem® XF Basal Medium containing MSC NutriStem® XF Supplement Mix (named “MSC NutriStem® XF Medium” Biological Industries, Cromwell, CT) supplemented with 5% (v/v) PLTGold before seeding at 5260 cells/cm^2^ in a CellBIND 24 well plate (Corning, Corning NY). After 4 days, media in the wells with cells assigned for adipogenesis were replaced with MSCgo™ Adipogenic Differentiation Medium (Biological Industries, Cromwell, CT) containing Adipogenic Differentiation Supplement Mix I and Adipogenic Differentiation Supplement Mix II (Biological Industries). Control cultures were maintained in MSC NutriStem® XF Medium. Cells were kept for six days in the respected media, changing media once, before adding to the cells fresh respected media containing 1:500 dilution of IncuCyte NucLight Rapid Red Reagent for Nuclear Labeling and 1:1000 dilution of LipidSpot™ 488 Lipid Droplet Stain (Biotium, Fremont, CA). After 30-min incubation at 37^0^C in a fully humidified incubator supplied with 5%CO_2_, plates were placed in an IncuCyte S3 Live Cell Analysis instrument for red nuclei count and green lipid droplets total green-integrated intensity (GCU) imaging using 20 × objective. Fluorescent images of red nuclei (imaged at 681 nm) and green lipid GCU (imaged at 585 nm) from sixteen fields in each well were captured every 6 h for 24 h. The data acquisition, visualization and analysis were done using internal IncuCyte S3 Analyzing Software. Each GCU value per well was normalized to the number of cells (red nuclei count) per well.

## Secretome analysis of resting aMSC

aMSC were seeded at 2105 cell/cm^2^ in 6 well pates with 2.5 ml PLGold media/well. Forty-eight hours later, media were replaced with 2 ml fresh media/well. One well kept with media only was used as a control background values. Four days (96 h) after, media were collected, spun for 5 min at 750 × g and supernatants store at -20^0^C until use. Immediately after collecting the media, 1 ml of fresh media containing 1:500 diluted IncuCyte NucLight Rapid Red Reagent was added to the cells. Cell number (red nuclei count) was counted in an IncuCyte S3 Live Cell Analysis instrument. For determining aMSC secretome content a 20plex custom made kit (Human Cytokine/Chemokine, Human Bone and Adipokine Magnetic bead panel, EMD Millipore, Burlington, MA), and Luminex xMAP technology (R&D Systems Inc., Minneapolis, MN) were used. The secretome assay was done in triplicate and was performed following the manufacturer's instructions. The plates were read by the MAGPIX instrument using xPONENT software for acquisition (Luminex, Austin, TX). Data analysis of the median fluorescence intensity (MFI) and coefficient of variance (CV%) estimation was done by MILLIPLEX Analyst 5.1 software (EMD Millipore). The analyte concentrations (pg/ml) were normalized to 1 × 10^6^ cells.

## Analysis of aMSC senescence status by droplet digital polymerase chain reaction (ddPCR)

For establishing the status of aMSCs senescence we developed a Droplet Digital PCR (ddPCR) protocol for estimating the transcription level of CDKN1, p16^INK4A^, p53 and RB1 cell cycle inhibitor markers. Total RNA from 1.5 × 10^6^ cell pellets was extracted using RNeasy Mini Kit (Qiagen, Germantown, MD). The reverse transcription reaction was performed with random, Oligo(dT)_20_, primers using iScript cDNA Synthesis Kit (Bio-Rad, Hercules, CA). For ddPCRs fluorescent-labeled custom-designed primers and probe for p16^INK4A^:

- p16^INK4A^ forward primer: 5’ GCC CAA CGC ACC GAA TAG 3’,
- p16^INK4A^ reverse primer: 5’ ACG GGT CGG GTG AGA GTG 3’ and
- p16^INK4A^ probe: FAM6-TCA TGA TGA TGG GCA GCG CC-TAMRAIowaBlack, (IDT, Coralville, IA) were used.

For the other tested cell cycle inhibitor markers as well as for TATA binding protein (TBP) as reference gene, commercially available fluorescent-labeled expression primers and probes were used (Bio-Rad).

The ddPCR setup was as previously described [36]. The final concentration of primers and probes in the reactions was 900 nmol/L and 250 nmol/L, respectively. Multiwall plates were sealed, vortexed briefly, centrifuged and placed on an automated droplet generator (AutoDG- Bio-Rad). Each sample was partitioned into 15,000–20,000 droplets. PCR amplification was performed on a Veriti Thermal Cycler (Applied Biosystems). The initial heating at 95 °C for 10 min was followed by 60 cycles of denaturation at 94 °C for 30 s, annealing and extension at 58 °C for 1 min, and a final extension step at 98 °C for 10 min. The completed reactions were stored at 4 °C until reading them on a QX200 droplet reader (Bio-Rad). Data analysis was performed using 2D Module of the QuantaSoft software (BioRad).

## Quantitative flow cytometry assay for immunoprofiling

### Procedural factors

An separate group of 15 IPF patients were identified from Interstitial Lung Diseases Outpatient Clinic by an expert pulmonologist in IPF and other fibrotic diseases of the lung following the ATS/ERS/JRS/ALAT Statement criteria [31]. All the aspects of this study involving samples from IPF patients and age- and gender-matched healthy volunteers were reviewed and approved by the Mayo Clinic Institutional Review Board. All subjects provided written informed consent to participate.YYY.

To characterize the circulating immune phenotype, peripheral blood samples from 87 healthy volunteers (30 of which age and gender matched) and from a separate group of 15 IPF patients were collected in K_2_EDTA tubes (Becton Dickinson, Franklin Lakes, NJ) at initial or return visits. Un-manipulated whole blood was stained with antibodies directly, within 12 h of collection as previously described [37, 38]. Quantitative flow cytometry was performed to comprehensively assess 110 leukocyte populations and phenotypes from lymphocytes, monocytes and granulocytes as previously described [37, 38]. The flow cytometry data were analyzed using Kaluza 2.1 software (Beckman Coulter), allowing quantification of the absolute number as well as percent of immune cell subtypes.

### Statistical analysis

Results are expressed as mean ± SD. Statistical analysis was performed using GraphPad Prism 8 software. Intergroup comparisons of parametrically distributed continuous data were made using un-paired two-tailed Student's *t-*test. Differences were considered significant when p values *p < 0.05, **p < 0.01, ***p < 0.001, ****p < 0.0001. Correlations between IPF patients pulmonary function tests (PFT) values were established by calculating the Pearson correlation coefficient (*r*). Flow cytometry data are either represented as percentage of population or number of cells/μl. ddPCR data and presented as mean of three with CV%.

## Results

### Isolation, propagation and identification of adipose MSC (aMSC)

**Fig. 1:**
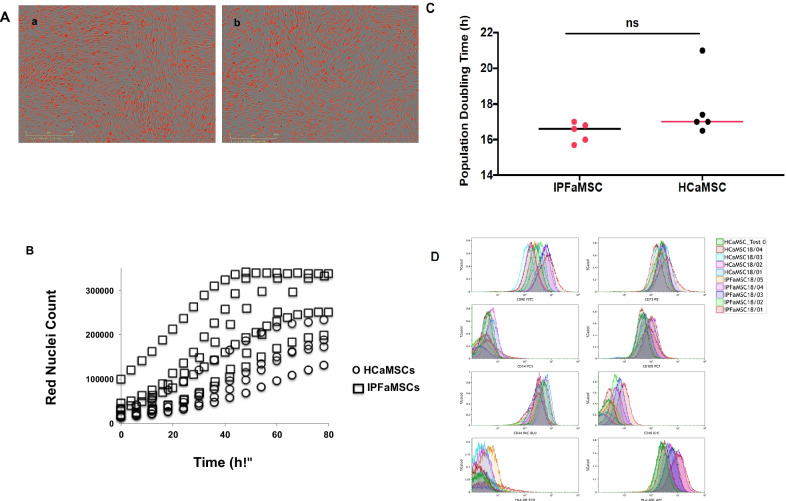
Proliferation, growth rate kinetic, doubling time comparison. **A**. Representative images of HCaMSC (*a*) and IPFaMSC (*b*) growth for 2 days in IncuCyte S3 Live Cell Analysis instrument. All tested cells lines exhibit spindle-shaped adherent morphology. Nuclei are stained red. Scale bar = 400 μm. **B.** HCaMSC and IPFaMSC cell proliferation estimated by counting the number of red nuclei for 3 days. **C.** Unpaired Student's t test analysis of population doubling times of both tested groups. Dots represent individual cell line values. Median values for IPFaMSC and HCaMSC were 17.0 and 16.6 h, respectively. Differences were considered significant when *p < 0.05, compared with control cell lines values. **D.** Flow cytometry results of IPFaMSCs HCaMSCs phenotypes. All ten cell lines tested more than 95% positive for typical MSC markers CD90, CD73, CD105, CD44 and HLA-ABC expression, and negative for lineage markers CD14, CD45 and HLA-DR expression

All tested cell lines (five IPFaMSC and five HCaMSC) expanded and grew as spindle-shaped adherent monolayer cells (Fig. 1A). There is no difference in proliferation rate as well as population doubling times between the cells from both tested groups: IPFaMSC and HCaMSC t_d_s were 16.4 ± 0.55 and 17.8 ± 1.83 h, respectively (Fig. 1B and 1C). Growth rate kinetic parameters are summarized in Additional file 1.

According to the criteria established by the International Society for Cellular Therapy [34] a flow cytometry protocol previously described [37, 38] was used to establish the aMSC identity and purity. All ten cell lines were consistent with MSC expression of classical MSC markers: CD44, CD73, CD90, CD105 and HLA-ABC and showed lack expression of lineage markers: CD14, CD45 and HLA-DR as summarized in Fig. 1D. No difference in expression of cell surface markers was noticed between aMSC isolated from IPF patients and HCs.

### Morphologic characterization

**Fig. 2:**
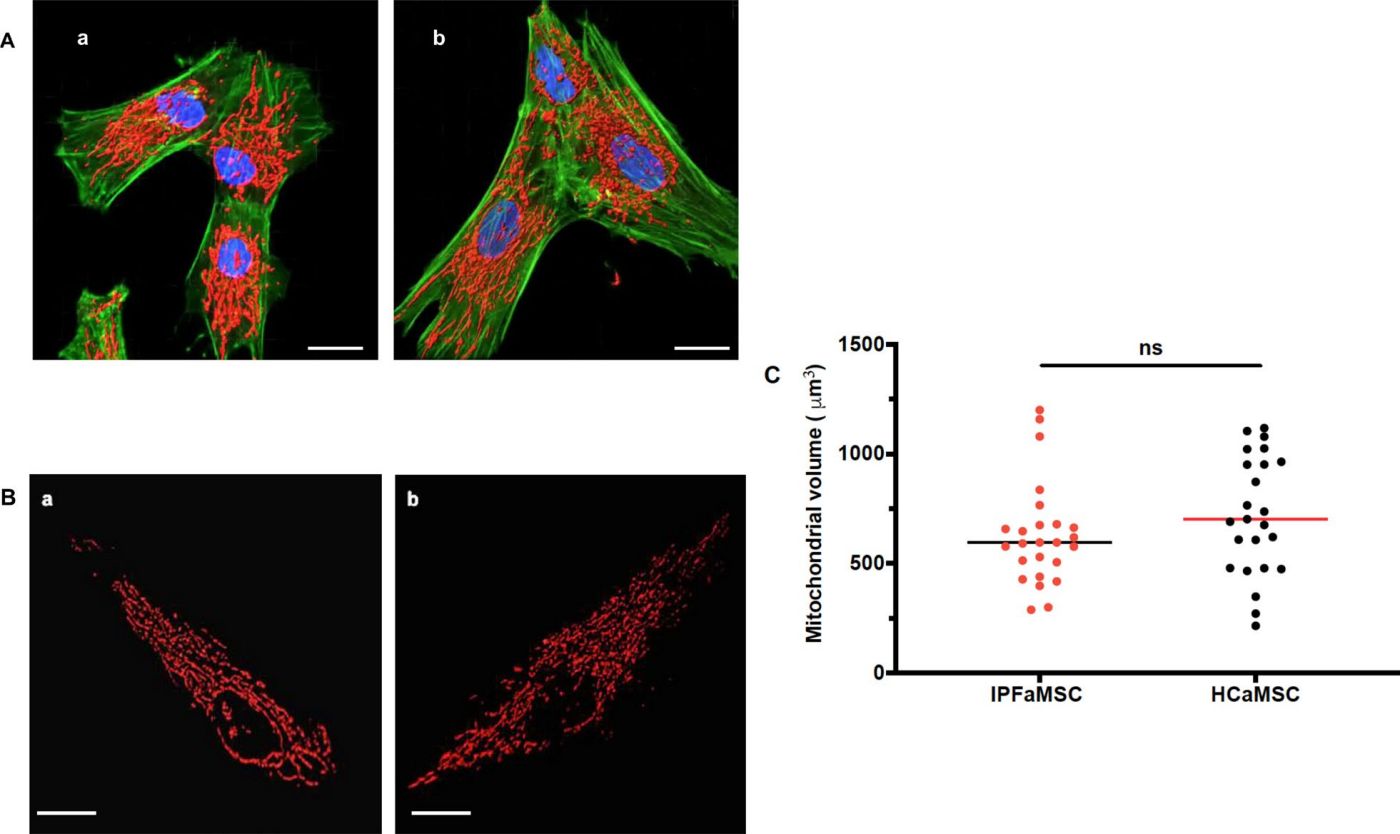
Cell morphology and mitochondrial volume evaluation by laser scanning confocal microscopy. **A** Representative fluorescence images of HCaMSC (*a*) and IPFaMSC (*b*). Scale bar = 20 μm. **B**. Representative images of mitochondria 3D isosurface in HCaMSC (*a*) and IPFaMSC (*b*) created by Imaris 8 software. Scale bar = 20 μm.** C** Statistical analysis of IPFaMSC and HCaMSC cells mitochondrial volumes was done by unpaired Student's t test. Dots represent individual cell values. No significant difference in the mitochondrial volume between two groups was found, with median values for IPFaMSC and HCaMSC of 596 and 702 μm^3^, respectively. Differences were considered significant when *p < 0.05, compared with control cell values

To evaluate cellular morphology and obtain more detailed information for the intracellular structures of tested aMSC, laser scanning confocal microscopy was used. Cells were stained with AlexaFluor™ 488 Phalloidin as actin F stain, MitoTracker Red CXMRos as mitochondrial stain and Hoechst 33342 to define cell nucleus. In both tested groups cells exhibit spindle-like morphology, branched cytoplasms and intact cytoskeletons. Mitochondria distribution is concentrated around the nucleus and along filopodia. Nuclei appear characteristically elliptical and uniformly speckled (Fig. 2A) which indicates homogeneous distribution of the chromatin throughout the nucleus. Using Z-stack confocal images mitochondria 3D isosurface was created, and the mitochondria volume was calculated using Imaris 8 software (Fig. 2B). Student's t test analysis of mitochondrial volumes in both groups showed no differences (Fig. 2C).

### Adipogenic differentiation

**Fig. 3:**
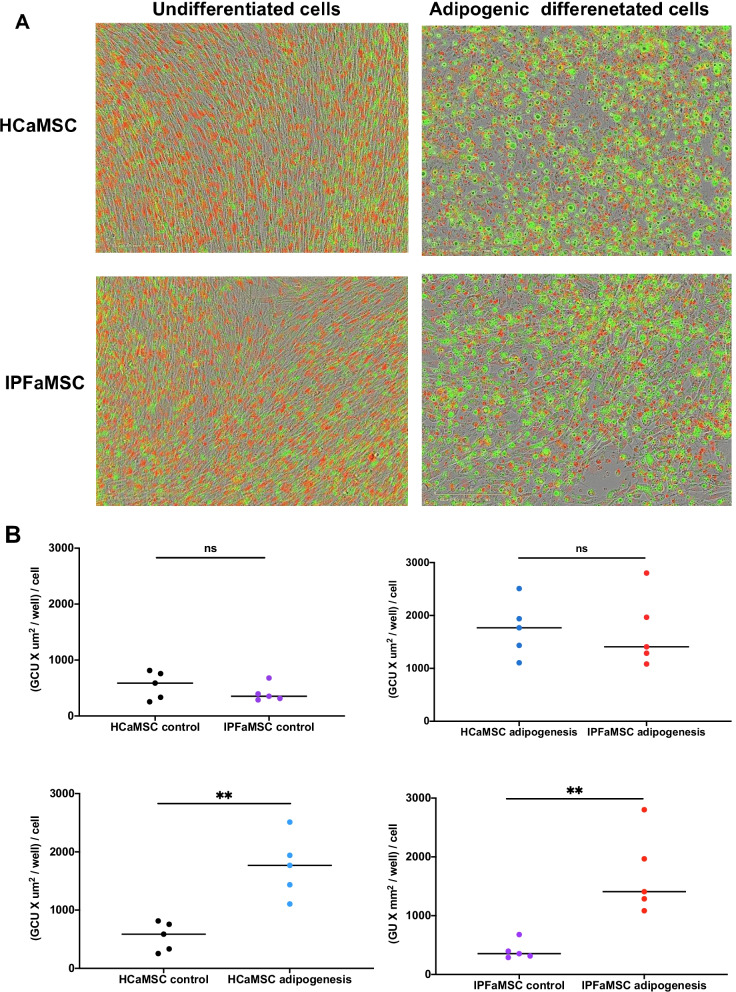
Adipogenic differentiation of IPFaMSCs and HCaMSCs. **A**. Representative images of undifferentiated and adipogenic differentiated (*a*) HCaMSC and (*b*) IPFaMSC. Scale bar = 200 μm. **B**. Unpaired Student's* t* test was used for analyzing the statistical differences of adipogenesis in undifferentiated HCaMSC and IPFaMSC. No significant difference in growth rate in nonadipogenic media between these two groups was found, with median values of 588 (GCU x mm^2^/well)/cell and 353 (GCU x mm^2^/well)/cell, respectively. No significant difference was found between adipogenic capacity of HCaMSC and IPFaMSC with median values of 1768 (GCU x mm^2^/well)/cell and 1408 (GCU x mm^2^/well)/cell, respectively. Significant difference was found between undifferentiated and adipogenic differentiated HCaMSC as well as between undifferentiated and adipogenic differentiated IPFaMSC. Dots represent individual cell lines values. **p < 0.01, compared with control cell lines

aMSC differentiation into adipocytes was confirmed by the presence of prominent green fluorescent lipid droplets compared to undifferentiated aMSC (Fig. 3A). aMSC from both groups showed no significant differences in adipogenic capacity (Fig. 3B) suggesting that cell lines from both groups have the similar adipogenic potential.

### Secretome analysis of resting aMSC

**Fig. 4:**
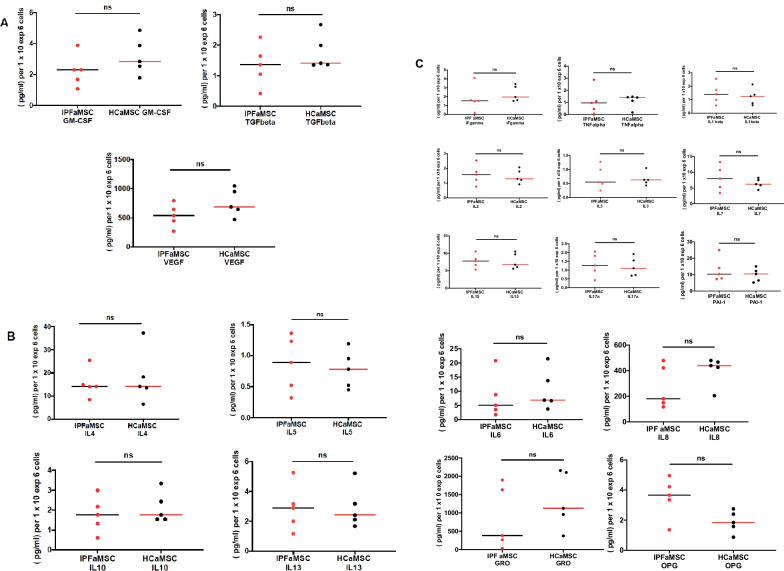
Unpaired Student's t test of the secretome profile of IPFaMSC and HCaMSC cells. The Luminex assay was performed in triplicate on media collected 4 days after initial incubation of the cells.** A** Secreted growth factors. ** B** Secreted anti-inflammatory cytokines. ** C** Secreted pro-inflammatory cytokines, group 1 (upper graphs)  and secreted pro-inflammatory cytokines, group 2 (lower graphs). CV% was less than 13% for all analytes. The concentrations of analytes (pg/ml) were normalized to the number of cells in each well and expressed as (pg/ml)/1 × 10^6^ cells. Data are expressed as mean and dots represent individual cell line values. Differences were considered significant when *p < 0.05

The functional capacity of aMSC from both groups was assessed by their secretome profile. The analysis of protein content of the aMSC secretome was performed on media collected 4 days after incubation with the cells. For base line, the concentrations of the assayed analytes in the media alone were used. The analytes were divided into three groups: growth factors (GM-CSF, TGF and VEGF, Fig. 4A), anti-inflammatory cytokines (IL-4, IL-5, IL-10 and IL13, Fig. 4B) and pro-inflammatory cytokines (IFN-γ, TNF-α, IL-1β, IL-2, IL-3, IL7, IL-15, IL17A, PAI 1, IL-6, IL-8, GRO and OPG, Fig. 4C). No differences in secretion of the analyzed cytokines were observed between the two tested groups. The concentrations of analytes (pg/ml) were normalized to the number of cells in each well and expressed as (pg/ml)/10^6^ cells. Student's t test analysis of the secretome in both aMSC tested groups showed no differences.

### Senescence status of aMSC

**Fig. 5:**
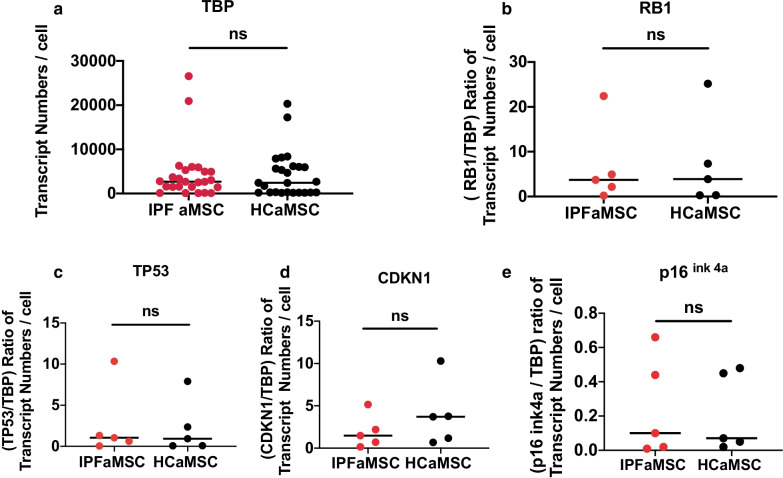
Senescence status of aMSC. Unpaired Student's t-test of cell cycle inhibitor markers transcript numbers. (**a**) The median values of TBP for IPFaMSC and HCaMSC were 2691 and 2447 transcript number/cell, respectively. (**b**) The median values of the normalized (RB1/TBP) ratio for IPFaMSC and HCaMSC were 3.72 and 3.89 transcript numbers/cell, respectively. (**c**) The median values of the normalized (TP53/TBP) ratio for IPFaMSC and HCaMSC were 1.04 and 0.93 transcript /cell, respectively. (**d**) The median values of the normalized (CDKN1/TBP) ratio for IPFaMSC and HCaMSC were 1.48 and 3.71 transcript number/cell, respectively. (**e**) The median values of the (p16^ink4a^/TBP) ratio for IPFaMSC and HCaMSC were 0.1 and 0.7 transcript numbers/cell. The CV% between triplicates was less than 15% for all tested cell cycle inhibitor marker transcripts. No significant difference between two tested cell groups was found in normalized transcript numbers of cell cycle markers inhibitors. Dots represent individual cell line values. Differences were considered significant when *p < 0.05, compared with control cell lines

The expression levels of the p16^INK4A^/Rb and p53/p21CIP1 (CDKN1) pathway members of the cells’ senescence phenotype were analyzed by the ddPCR method, a sensitive and accurate method of measuring small changes in gene expression [41, 42]. The expression level of TBP was measured along with each cell cycle inhibitor marker and used for normalization of the expression levels of the genes of interest. The normalized transcript values were expressed as a ratio of genes of interest transcript copy number to TBP transcript copy number per cell. Unpaired Student's t test showed that there is no significant difference in the expression levels between the two aMSC tested groups (Fig. 5).

### Quantitative flow cytometry assay for immunoprofiling

**Fig. 6:**
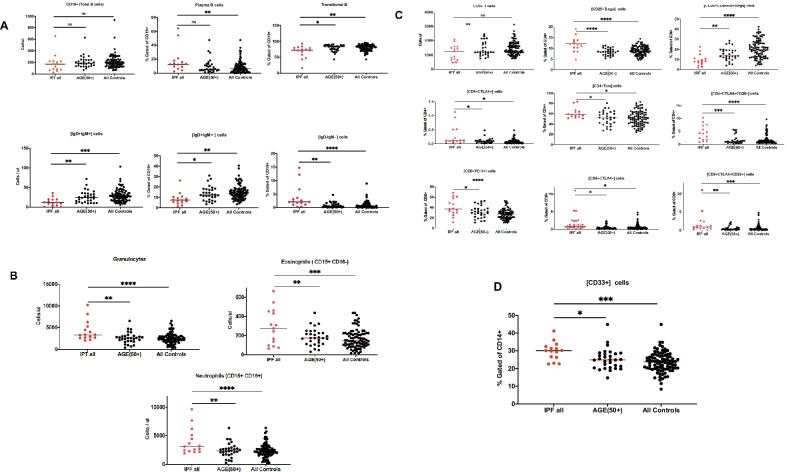
Immnunoprofile comparison of blood samples from IPF patients and HCs using unpaired Student's t test. **A**. B cells panel. There is no difference in total B cell numbers between the tested groups. IPF patients have decreased percentage as well as cell number of [IgD + IgM +] cells, smaller percentage of transitional B cells, while higher percentage of [IgD-IgM-] cells. **B**. Granulocytes, eosinophils and neutrophils panel. IPF patients have elevated numbers of granulocytes, eosinophils as well as neutrophils than HCs. **C**. T cells panel: Total number of T (CD3 +) cells is not different between two groups, while the percentages of [CD4 + CTLA4 +], CD4 + CTLA4 + CD28 +], [C8 + CTLA4 +], [C8 + CTLA4 + CD28 +], [CD8 + PD-1 +] cells as well as [CD25 + Treg] and [CD4 + Tcm] T cells are elevated. Only the percentage CD25 + CD45RA + Treg cells is decreased. **D**. Monocytes panel**:** The monocyte phenotype showed higher percentage in IPF patients. Dots represent each individual values. *p < 0.05, **p < 0.01, ***p < 0.001, ***p < 0.0001, compared with control group

Multi-parameter flow cytometry was conducted of whole blood samples from 15 IPF patients and 87 HCs between age 19 and 69 with 30 of those being 50 + years old (Table 1). Seven leukocyte phenotypes were measured as cells/μl and as a percentages of larger groups: granulocytes, [CD14 +] monocytes, [CD19 +] B cells, [CD56 + CD16-] NK cells, [CD3 + T] cells, [CD4 + T] cells, [CD8 + T] cells, and [CD3 + CD56 +] cells. Out of 110 leukocyte phenotypes tested, 16 were found different in IPF patients from HCs. Although there was no difference between IPFs and HCs samples in the total percentage of plasma B cells, as well as the number of (cells/μl) of [CD19 +] B cells as a group the subsets of [CD19 +] B cells were different. IPF patients exhibited lower percentages of transitional B cells. The number of (cells/μl) and percentages of [CD19 +] naïve [IgD + IgM +] cells in IPF were also lower than in HCs. At the same time the percentage of double-negative [IgD-IgM-] B cells were elevated in IPF patients (Fig. 6A and Additional file 2). IPF patients exhibited elevated cell counts (cells/μl) of granulocytes, eosinophils, and neutrophils compared to HCs (Fig. 6B and Additional file 2). The total number of [CD3 + T] cells between IPF patients and HCs was not different, but the subpopulations of the T cells in IPF patients were (Fig. 6C and Additional file 2). The percentage of [CD25 + CD45RA + Tregs] cells decreased in IPF patients, while the percentages of [CD25 + Tregs], [CD4 + Tcm], [CD8 + PD-1 +] were increased. Percentages in T signaling cells [CD4 + CTLA4 +], [CD4 + CTLA4 + CD28 +], [CD8 + CTLA4 +] and [CD8 + CTLA4 + CD28 +] in IPF patients were also noted as well as an increase in percentage of [CD33 +] monocytes in IPF patients (Fig. 6D and Additional file 2).

**Table 1:** Demographic characteristics and lung function indices

Variable	Characteristics	IPF patients (*n* = 15)	Healthy control	Healthy control
			Age 50 + (*n* = 30)	All (*n* = 87)
Age (years)	Mean ± STD	76 ± 6.94	56 ± 3.74	40 ± 13.32
	Range	65–89	50–69	19–69
Gender	Female	6	7	20
	Male	9	23	67
Lung function				
FVC% predictive		73 ± 15.6		
FEV1% predictive		85.2 ± 14.1		
VC_max_ % predictive		74.2 ± 15.1		
Treatment at immunophenotyping (*n*)				
Pirfenidone		5		
Nintedanib		2		
Prednisone		1		
No medications		7		

Data are presented as mean ± SD, FVC% predictive = % of Forced Vital Capacity; FEV1% predictive = % of Forced Expiratory Volume in the 1st second, predictive; VC_max_ % predictive = % Maximal Vital capacity

“Predictive” means values adjusted for patient age, gender and race

**Fig. 7:**
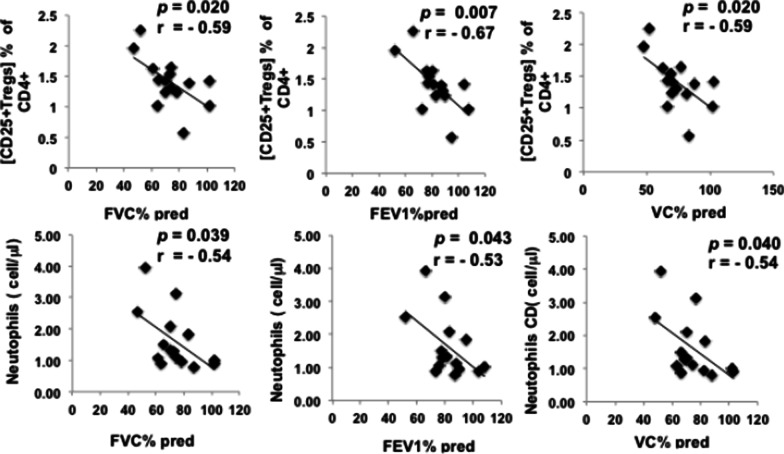
Pearson’s correlation coefficients (**r**) for IPF phenotype = f (pulmonary function test). Dots represent each individual values. Correlation graphs show Pearson correlation coefficient **r**- and *p*- values. *p*-values were determined by Student’s *t* test distribution for Pearson correlation. Lines represent the best fit resulting from linear regression analysis. The results are significant at *p < .05

To determine whether any of these identified differences in IPF immunophenotypes were associated with IPF patient’s pulmonary functions, ratios of immunophenotypes for each subject (IPF patients and HCs, age 50 +) were calculated by dividing individual leukocyte phenotypes with the mean of the age 50 + cohort HCs and performed a Pearson’s correlation coefficients tests (Fig. 7). Pulmonary Functional Tests (PFT) were performed on the same day as blood draw collection for immunoprofiling with the exception of two patients which had a gap of a few months between the PFT and blood draw. Three phenotypes, [CD25 + Treg] cells neutrophils showed moderate inverse correlation with the PFTs (Fig. 7). Treg cells had the highest degree of inverse correlations with all PFTs values and have the highest degree of probability being correlated with IPF.

### Potential medications effect

To exclude the influence of potential medications effect on IPF patient’s immunophenotyping, ad hoc statistic comparisons were made. No difference in the percentage or number of measured phenotypes was found to be affected by patient’s medications.

## Discussion

In this study, we provide data that autologous adipose MSC from IPF patients retain all tested potential mechanisms of action of therapeutic MSC as therapeutic cells as well as baseline identification of immune biomarkers for IPF. MSC’s mode of action is still not fully understood, but they target the sites of injury, enhance angiogenesis, modulate immunity and contribute to epithelial tissue repair [19–23, 26, 43]. Promising results of preclinical studies using MSCs suggest that they may represent a potential therapeutic option for the treatment of chronic lung diseases including IPF [24–26]. Few clinical trials have been reported using adult allogeneic MSCs of different sources for treatment of IPF. Tzouvelekis et al. conducted a phase 1b clinical trial to assess the safety of the allogeneic adipose MSCs in treatment of IPF [27]. Chambers et al. reports using placental MSCs in a clinical 1b study on a small cohort of IPF patients [28] . Glassberg et al. were using bone marrow-derived MSCs in a phase I safety clinical trial [29] . Averyanov et al. reports that in phase I/IIA clinical study high cumulative doses of bone marrow MSCs were used [30]. All of the clinical trials so far demonstrate that use of MSCs in IPF treatment is safe and well tolerated, even in the case when an extremely high dose of MSCs was used [30].

Subcutaneous adipose tissues MSCs are frequently used for clinical applications because of their easy access, minimal invasive procedure and their therapeutic potentials [32, 44]. This study examined the characteristics of adipose aMSCs isolated from IPF patients (IPFaMSC) and age- and gender-matched healthy controls (HCaMSC) to establish their potential suitability for IPF treatment.

Comprehensive characterization of aMSCs from both groups was carried out using a combination of flow cytometry, ddPCR, confocal microscopy, IncuCyte Live Cell Imaging and LUMINEX xMAP technologies. Our data provide evidence that autologous adipose tissue-derived MSCs from IPF patients exhibit the same properties as the adipose tissue-derived MSCs from the HC. In summary, the aMSCs were adherent to plastic and exhibited the same small, spindle shape morphology, all tested cell lines had characteristic MSC phenotype [34, 39, 40], the growth curves of IPFaMSC and HCaMSC cell lines were identical and in agreement with the general behavior of the MSCs including exponential growth [45], and all aMSCs display similar mitochondrial morphology and assessing mitochondrial volumes. Changes in mitochondrial volume have been associated with a wide range of important biological functions and pathologies [46]. In particular, current evidence suggests that the areas of fibrosis seen in IPF patients lungs share many mitochondrial dysfunction features [47]. The mitochondria of both tested cell lines have a typical shape and distribution for healthy MSC mitochondria [48]; also, the IPFaMSC mitochondrial volume is not different from mitochondrial volume of HCaMSC, which indicate that IPFaMSC are healthy, not in a state of stress and are fully functional. The capacity and plasticity for adipogenic differentiation of IPFaMSC were the same as of HCaMSC also suggestive of a stem cell phenotype. MSC are known to interact and actively communicate with their surrounding microenvironment through the secretion of cytokines and growth factors. Both cell groups secrete the same amount of growth factors, anti-inflammatory as well as pro-inflammatory cytokines, which indicates that the cell lines from both groups have the same capacity for regulating the tissue regeneration, proliferation, angiogenesis and modulation of inflammation. Finally, it has been long recognized that cellular senescence significantly contributes to the aging-related declines in tissue regeneration capacity and in the pathogenesis of aging-related diseases, such as IPF. The mechanism of how senescent cells contribute to aging and aging-related diseases remains unclear. Resident stem cells are particularly sensitive to senescence stresses. One hypothesis of the pathogenic fibrosis of IPF is that cellular senescence leads to exhaustion of the resident stem cells, which, in turn, causes a decline in tissue regenerative capacity during aging or upon injury [49]. Our data are representative of the transcriptome and would need to be confirmed with follow-up of protein analysis. But taken together, these findings suggest that IPF patients’ aMSC are as capable and functional as healthy volunteers’ aMSC. Also, if the pathogenesis of IPF includes local depletion of MSC, the patient could be a reasonable resource for replacing that deficit.

While establishing the suitability of using autologous aMSCs for IPF treatment, we also wanted to find out whether a known mechanism of action of MSC therapy (that is an altered immune system) is associated with the disease pathology. To do so, we assessed the immune status of IPF patients by quantifying their circulating phenotypes and compared them with healthy controls. In addition, we sought to identify changes in IPF patient’s immunological phenotypes, which correlate with their lung function indices.

Circulating granulocytes, neutrophils and eosinophils were found elevated in IPF patient blood. These cells are first to respond to the presence of pathogens in human lungs or upon tissue damage. They migrate from periphery to the damaged lungs in response to secreted chemokine and interleukin signals from invaded lungs and become activated [48, 49]. They were found elevated in bronchoalveolar lavage fluid (BALF) and sputum of IPF patients [50], as well as in peripheral blood, sputum and BALF in chronic obstructive pulmonary disease (COPD) patients [11]. Neutrophil count in IPF patients in our study inversely correlates with all three measured pulmonary indices. High levels of neutrophil elastase were found in lung parenchyma and also in both BALF and IPF patient serum. Therefore, neutrophils might indeed play an important role in the pathogenesis of IPF [52]. The same result was found in IPF patients BALF along the increased IL-8 concentrations [51], and it was speculated that those findings might be predictive for future exacerbations of IPF [53]. There is evidence that neutrophils might promote fibrosis via their regulation of extra cellular matrix (ECM) turnover [52, 54, 55]. Kinder et al. report that increased number of neutrophils in BALF is associated with early mortality in IPF [56]. All these effects/interactions are complex and multifaceted, and they are not fully studied nor understood in IPF.

Recent studies have shown that B cells, as part of adaptive immunity, are involved in IPF pathology [13, 14, 58]. In our study the total number of B cells and the percentage of Plasma B cells were not different between two tested groups. Our measurement of circulating [CD27 + IgM−IgD−] cell percentage shows an increase in IPF patients. It has been found that stimulated [CD27 + IgM−IgD−] cells activate telomerase and are responsible for age-related exhaustion of the B cells [16, 59, 60]. Since immune exhaustion and telomerase dysfunction have been implicated in IPF pathology, this subset of B-cells may be a good topic for further research.

In contrast to [CD27 + IgM−IgD−] cells, the number of transitional B cells as well as the number and percentage of [CD27 + IgM + IgD +] cells in the IPF patients’ blood was lower than in HCs. Characterization of pathological pattern in lung tissue samples from IPF patients [14, 15] has shown that there are lymphoid aggregates present in the regions with dense scars. Gene expression profile of the same lung tissue areas has identified a cluster of overexpressed genes of which the chemokine (C-X-C motif) ligand 13 (CXCL13) was elevated in comparison with the control group. It is known that CXCL13 selectively mediates B-cell migration. As immature B cells emigrate from the bone marrow and enter the blood stream, they migrate toward the wounded organ (the lungs in the case of IPF), being attracted by secreted CXCL13 chemokine from the wounded lungs [13–15], where they are forming the lymphoid aggregates. This model supports our findings of decreased circulating transitional B cell percentage and [CD27 + IgM + IgD +] cells in IPF patients’ peripheral blood. It is worth noting that the longitudinal studies of CXCL13 concentration in peripheral blood of IPF patients, but not in COPD patients, were also found elevated and were correlated with IPF survival [14, 15], which may be of use as an indicator for severity of the disease. Reduced amounts of these B cells subpopulations also have been found in the blood of sarcoidosis patients [61] and in patients with common variable immunodeficiency (CVID) with recurrent lower respiratory tract infections [62]. Studying the functional capacity of these cells in early inflammatory responses Seifert et al. concluded that [CD27 + IgM + IgD +] memory B cells are generated in T cell-dependent immune response [61]. The exact role of this B cell subpopulation in IPF pathology is not yet clear.

The role of T cells in the pathology of pulmonary fibrosis is poorly understood and controversial. It was hypothesized that if IPF progression depends on an adaptive immune component, it would be possible to find associations between phenotypic changes of circulating T cells and clinical manifestations of the disease [57]. Current evidence suggests that there are substantial T cell subset abnormalities in the blood [63–65], peripheral blood proteome profile [65], BALF [66, 67] and lung tissue in IPF patients [68, 69] which may contribute to the fibrotic processes**.** In our study, the total number of circulating T cells in IPF patients does not differ from HCs, but there is a difference in T helper cells as well as in cytotoxic/suppressor T cell subpopulations. Five [CD4 +] subpopulation T helper cells have increased percentages in comparison with HCs of which [CD4 + CD25 + Tregs] are most studied. Patients with IPF had larger fractions of these circulating cells, and they are inversely correlated with all three measured pulmonary indices. Our results are in agreements of findings of Hou et al. [63]. An increase in circulating [CD4 + CD25 + Tregs] cells is a hallmark of disturbed immune homeostasis in various pulmonary diseases, including IPF [70]. A lower proportion of regulatory [CD25 + CDRA + Treg] cells in IPF patients in our study are in agreement with the findings in other studies where low proportion of these cells have being detected in blood and BALF of IPF patients [63, 71]. A low percentage of regulatory T cells have limited inhibitory activity and hence impaired immune tolerance [71].

The involvement of T helper and cytotoxic/suppressor cell subsets in pathology of IPF has been poorly understand (76). The increased percentage of [CD4 + CTLA4 +], [CD4 + CTLA4 + CD28 +], [CD8 + PD-1 +], [CD8 + CTLA +] and [CD8 + CTLA4 + CD28 +] indicate that there is substantial downregulation in immune response in IPF patients [72].

Monocytes are known to contribute to the pathogenesis of idiopathic pulmonary fibrosis as was shown in the retrospective, multicenter cohort study [73]. The robust association of high monocyte count associated with mortality in other fibrotic diseases suggests they might contribute to the pathogenesis of these diseases as well. In our study IPF patients have a higher percentage of circulating [CD33 +] monocytes, which may contribute to the progression of the disease.

## Conclusion

MSC-based therapies for IPF, so far, are using allogeneic cells for treatment. Although the safety and tolerance of their use has been confirmed, it is still not clear whether these are the optimal source for therapy in IPF. Autologous adipose MSC are suitable for multiple doses potentially even the ability of dosing to a therapeutic response. Our study shows that adipose MSCs from IPF patients are not part of IPF pathology, are fully functional in all of our assessments and may be used for IPF therapy.

Understanding the immunological status of IPF patients may provide insight into their immunity and its role in etiology of the disease. To our knowledge, our study is the most comprehensive evaluation of circulating phenotype in IPF patients so far and provides further evidence for the role of immunity in the pathogenesis of IPF. The increased Tregs in the IPF patient’s peripheral blood correlate inversely with disease severity. Treg subpopulations may be promising prognostic factors for IPF. Characterization of the peripheral immune phenotypes in IPF patients may answer the question whether or not the immune events identified in the circulation can be used as a monitor for personalized IPF therapy, to potentially classify or stage the disease, and identify those more likely to respond to therapy. Additional studies are needed with an expanded cohort of patients for positive identification of circulating phenotypes from peripheral blood as potential biomarkers for IPF.

## Supplementary Information

**Additional file 1**. Growth rate kinetic, population doubling time and *R*^2^ for the linear regressions. *k* represents cell growth rate per hour; *t*_*d*_ represents cell doubling time per hour, and R^2^ represents the coefficient of determination for the linear regression.

**Additional file 2**. Comparison of immunophenotypes between healthy controls (HC, age 50 +) and IPF patients. In Blue: decreased mean values in IPF patients; in Red: increased mean values in IPF patients. *p < 0.05, **p < 0.01, ***p < 0.001, ***p < 0.0001, compared with control group. The phenotypes which correlate with the pulmonary functions are denoted with a red asterisk.

## Data Availability

Data from this manuscript are available upon request from the corresponding author.

## Abbreviations

aMSCs: Adipose tissue-derived mesenchymal stem cells
AO: Acridine orange
ATS/ERS/JRS/ALAT: American Thoracic Society/ European Respiratory Society/ Japanese Respiratory Society/ Latin American Thoracic Society
CDKN1/p21CIP1: Cyclin-dependent kinase inhibitor 1
COPD: Chronic obstructive pulmonary disease
CV%: Coefficient of variance %
CVID: Common variable immune deficiency
ddPCR: Droplet digital polymerase chain reaction
CXCL13: (C-X-C motif) ligand 13
ECM: Extracellular matrix
FEV1: Forced expiratory volume during first second
FVC: Forced vital capacity
GCU: Total green-integrated intensity
HCs: Healthy controls
HCaMSCs: Healthy controls adipose mesenchymal stem cells
IPF: Idiopathic pulmonary fibrosis
IPFaMSCs: Idiopathic pulmonary fibrosis patients adipose mesenchymal stem cells
MFI: Mean fluorescent intensity
MEM: Modified Eagle medium
MSCs: Mesenchymal stem cells
p16^ink4a^: Cyclin-dependent kinase inhibitor 2A
NK: Natural killer cells
p53: Cellular tumor antigen p53
PBS: Phosphate-buffered saline
PFT: Pulmonary function test
PI: Propidium iodide
PLGold: Human platelet lysate
PCR: Polymerase chain reaction
K_2_EDTA: Di-potassium ethylenediaminetetraacetic acid
RB1: Retinoblastoma transcriptional corepressor 1
RNA: Ribonucleic Acid
SD: Standard deviation
TBP: TATA binding protein
VC_max_: Maximal vital capacity
