# Supplementary material for: Normal ex vivo mesenchymal stem cell function combined with abnormal immune profiles sets the stage for informative cell therapy trials in idiopathic pulmonary fibrosis patients
Source: Stem Cell Res Ther. 2022 Jan 31;13:45. doi: 10.1186/s13287-021-02692-0 (PMC8802496; doi:10.1186/s13287-021-02692-0)
Supplement: Supplementary file 1 — Additional file 1. Growth rate kinetic, population doubling time and R2 for the linear regressions. k represents cell growth rate per hour; td represents cell doubling time per hour, and R2 represents the coefficient of determination for the linear regression. [file 13287_2021_2692_MOESM1_ESM.pdf]

Red Nuclei Count			
Sample	k (growth rate/h)	t_d (h)	R^2
IPFaMSC18/01	0.0418	16.6	0.99133
IPFaMSC18/02	0.0407	17.0	0.99130
IPFaMSC 18/03	0.0431	16.0	0.99387
IPFaMSC18/04	0.0441	15.7	0.99938
IPFaMSC18/05	0.0413	16.8	0.99431
HCaMSC 18/01	0.0408	17.0	0.99856
HCaMSC 18/02	0.0407	17.0	0.99411
HCaMSC18/03	0.0421	16.5	0.99838
HCaMSC18/04	0.0332	21.0	0.99893
HCaMSC Test 0	0.0399	17.4	0.99001
