# Supplementary material for: Normal ex vivo mesenchymal stem cell function combined with abnormal immune profiles sets the stage for informative cell therapy trials in idiopathic pulmonary fibrosis patients
Source: Stem Cell Res Ther. 2022 Jan 31;13:45. doi: 10.1186/s13287-021-02692-0 (PMC8802496; doi:10.1186/s13287-021-02692-0)
Supplement: Supplementary file 2 — Additional file 2. Comparison of immunophenotypes between healthy controls (HC, age 50 +) and IPF patients. In Blue: decreased mean values in IPF patients; in Red: increased mean values in IPF patients. *p < 0.05, **p < 0.01, ***p < 0.001, ***p < 0.0001, compared with control group. The phenotypes which correlate with the pulmonary functions are denoted with a red asterisk. [file 13287_2021_2692_MOESM2_ESM.pdf]

	IPF			HCs (age 50+)			2-tailed Student t-test	
Phenotype	Minimum	Maximum	Mean	Minimum	Maximum	Mean	p value	Sigificans
CD19+ total B cells, cells/μl	27	6552	180	69.96	629.17	213	0.3513	no
[Plasma B] % gated of CD19+	2.56	64.20	18.16	1.67	45.51	10.06	0.0561	no
[Transitional B] % gated of CD19+	15.76	92.84	67.55	60.35	90.66	78.86	0.0153	yes (*)
[IgD+IgM+] % of CD19+	1.42	26.25	8.42	4.34	30.94	13.64	0.0161	yes (*)
[IgD+IgM+] % of CD19+ (cells/ul)	1.13	36.16	13.45	9.99	72.00	26.52	0.004	yes (**)
[IgD-IgM-] % of CD19+	1.13	14.73	3.74	0.06	4.73	0.97	0.0014	yes (**)
Granulocytes (cell/ul)	2053	10217	4263	403	6589	2641	0.004	yes (**)
Eosinophils (CD15+CD16-) cells/ul	68	665	290	32	437	187	0.016	yes (*)
Neutophils CD15+CD16+ (cell/ul)	1954	9670	3982	245	6361	2455	0.0059	yes (**)
CD3+ T cells (cells/ul)	463	2088	1171	752	2443	1406	0.1422	no
[CD25+CD45RA+Tregs] % gated of CD4+	2.14	22.73	9.588	4.21	28.58	15.25	0.0049	yes (**)
[CD25+Tregs] % gated of CD4+	8.81	19.01	11.84	5.3	11.3	8.424	<0.0001	yes (****)
[CD4+ Tcm] % CD4+	51.09	83.71	61.01	27.26	67.11	51.93	0.0297	yes (*)
[CD8+PD-1+] % gated of CD8+	11.92	68.48	40.65	11.37	52.85	31.76	0.0425	yes (*)
[CD4+CTLA4+] % gated of CD4+	0.04	1.13	0.2813	0.02	0.48	0.1087	0.0126	yes (*)
[CD4+CTLA4+CD28+] % gated of CD4+	1.04	10.35	4.31	0.17	5.68	1.618	0.0004	yes (***)
[CD8+CTLA4+] % gated of CD8+	0.01	0.24	0.08733	0.01	0.16	0.02633	0.0122	yes (*)
[CD8+CTLA4+CD28+] % gated of CD8+	0.11	11.02	1.978	0.04	1.88	0.5467	0.0099	yes (**)
[CD33] % gated of CD14+	22.59	36.08	29.79	14.75	44.9	25.26	0.0114	yes (*)
